# Wild Boar as a Sylvatic Reservoir of Hepatitis E Virus in Poland: A Cross-Sectional Population Study

**DOI:** 10.3390/v12101113

**Published:** 2020-09-30

**Authors:** Iwona Kozyra, Artur Jabłoński, Ewelina Bigoraj, Artur Rzeżutka

**Affiliations:** 1 Department of Food and Environmental Virology, National Veterinary Research Institute, Al. Partyzantów 57, 24-100 Puławy, Poland; iwona.kozyra@piwet.pulawy.pl (I.K.); ewelina.bigoraj@piwet.pulawy.pl (E.B.); 2 Department of Large Animal Diseases and Clinic, Warsaw University of Life Sciences, Nowoursynowska Street 100, 02-797 Warsaw, Poland; artur_jablonski@sggw.edu.pl

**Keywords:** wild boar, hepatitis E virus, serosurvey, infection prevalence

## Abstract

The most important wildlife species in the epidemiology of hepatitis E virus (HEV) infections are wild boars, which are also the main reservoir of the virus in a sylvatic environment. The aim of the study was a serological and molecular assessment of the prevalence of HEV infections in wild boars in Poland. In total, 470 pairs of samples (wild boar blood and livers) and 433 samples of faeces were tested. An ELISA (ID.vet, France) was used for serological analysis. For the detection of HEV RNA, real-time (RT)-qPCR was employed. The presence of specific anti-HEV IgG antibodies was found in 232 (49.4%; 95%CI: 44.7–54%) sera, with regional differences observed in the seroprevalence of infections. HEV RNA was detected in 57 (12.1%, 95%CI: 9.3–15.4%) livers and in 27 (6.2%, 95%CI: 4.1–8.9%) faecal samples, with the viral load ranging from 1.4 to 1.7 × 10^11^ G.C./g and 38 to 9.3 × 10^7^ G.C./mL, respectively. A correlation between serological and molecular results of testing of wild boars infected with HEV was shown. HEV infections in wild boars appeared to be common in Poland.

## 1. Introduction

Hepatitis E virus (HEV) belongs to the *Hepeviridae* family and causes infections in humans and animals. Based on the genome analysis of different HEV strains, eight virus genotypes (gt) were identified [1]. Considering the similarities and differences in the genome structure of particular virus strains, so far, 36 virus subtypes have been distinguished [1]. Special attention is given to the zoonotic HEV strains assigned to gt 3 and 4, which also are responsible for cases of foodborne zoonoses [2,3,4,5]. In Europe, infections in livestock and wildlife are primarily caused by HEV gt 3 strains [6,7,8].

The most important wildlife in the epidemiology of HEV infections in a sylvatic environment are wild boar and several deer species [9,10,11]. The preliminary results of the occurrence of HEV infections in wild-living animals in selected regions of Poland confirmed infections only in wild boars [12,13]. A relationship was observed between the frequency of infection and the density of animals in a particular region. A phylogenetic analysis of the ORF2 genome fragment of Polish wild boar HEV strains confirmed their affiliation to gt 3 [13]. Meanwhile, the results of these studies provide evidence that HEV circulates in wild boars in Poland, creating a potential risk of virus transmission to farm animals and humans. Nevertheless, the pathogenesis of the disease in wild boars is still relatively poorly understood; however, it can be assumed that, as with pigs, it has an asymptomatic course [14,15]. The virus has been found in the liver, muscles and blood of infected animals [9,16]. The liver is the main site of HEV replication, which has been evidenced by histopathological lesions present in the livers of infected pigs [17] and other animals susceptible to infection [18,19,20]. In addition, extrahepatic sites of virus replication have been shown. HEV also efficiently replicates in the spleen, and to a lesser extent in the kidneys, lungs and lymph nodes [21,22,23]. As with pigs, a periodic shedding of the virus in the faeces of wild boars has also been observed [24].

The aim of the study was the serological and molecular assessment of the prevalence of HEV infections in wild boars in Poland.

## 2. Materials and Methods

### 2.1. Viruses

The HEV gt 3 strain of pig origin (accession number: MT770754) was used for the preparation of an RNA standard for determining the viral genome copy number in the animals’ livers and faeces.

### 2.2. Sera, Livers and Faeces of Wild Boar

In total, 470 pairs of samples (wild boar blood and livers) were analysed for the presence of virus-specific antibodies and HEV RNA, respectively. Additionally, 433 samples of faeces of wild boars were tested. The animals were hunted between 2015 and 2019 in 51 forest inspectorates belonging to 17 Regional Directorates of State Forests (RDSFs) in Poland. Samples were taken from juvenile (<2 years of age, 15–70 kg) and adult (>2 years of age, >70 kg) animals. The number of sampled wild boars was proportional to their population size inhabiting each RDSF (personal comm. General Directorate of the State Forests). Every 1000 animals of the population were represented by at least 1 sampled wild boar. The exception was RDSF Radom, from which 9 animals instead of 10 were sampled. Taking into account the size of the animal population present in the particular RDSFs, each RDSF was assigned to one of the following groups: large (20,000–30,000), medium (10–20,000) or small (<10,000). Blood samples (~75 mL) were taken from the main vein trunks or the animal’s chest cavity. During the evisceration stage, livers (~200 g) and faeces (~50 g) were also collected. The material was sent in refrigerated conditions to the laboratory. Upon arrival, the livers and faeces were kept frozen at −20 °C until use, while the blood was processed to obtain sera. The hunting of animals respected the Polish laws of animal protection. Detailed information on the number of tested animals and their origin is presented in Table 1.

### 2.3. Preparation of the HEV RNA Standard

Amplification of the ORF3 genome fragment of HEV gt 3 pig strain was performed by a real-time (RT)-PCR method described by Jothikumar [25]. The obtained 70 bp amplicons were purified from a 1.7% agarose gel using a QIAquick^®^ Gel Extraction Kit (Qiagen, Hilden, Germany) according to the manufacturer’s instructions. The DNA was cloned into a plasmid vector (pDrive Cloning Vector, Qiagen, Hilden, Germany) followed by its transfection into the competent *Escherichia coli* cells. Plasmid DNA containing the fragment of HEV ORF3 was transcribed using a T7 RNA polymerase (Riboprobe^®^ Combination Systems—SP6/T7, Promega, Madison, WI, USA). Subsequently, an RNA transcript was purified, quantified and diluted to obtain a concentration of 1 × 10^5^ HEV genome copies (G.C.)/µL.

### 2.4. Detection of Anti-HEV Antibodies in Wild Boars

Sera were obtained after centrifugation of coagulated blood at 4 °C, 1000× *g* for 10 min and subsequently frozen at −20 °C until use. The ID Screen^®^ Hepatitis E Indirect Multi-species ELISA kit (ID.vet, Grabels, France) was used to detect the presence of anti-HEV IgG antibodies according to the manufacturer’s instruction. The border cut-off values (S/P%) that allowed the sample to be considered as positive, doubtful or negative were calculated using the following formula: (optical density (OD) value of tested sample/OD value obtained for positive control) × 100%. The serum was considered positive when its cut-off value exceeded the borderline seropositivity of 70%. When the value ranged from 60% to 70%, it indicated a doubtful result. For negative sera, OD values were below 60%. The correct performance of the method was monitored using the results obtained for positive and negative control sera supplied with the assay.

### 2.5. Detection of HEV RNA Using Real-Time RT-qPCR

Isolation of HEV RNA from 100 µL of liver homogenate supernatant or 10% PBS-faecal suspension was performed using the QIAamp^®^ Viral RNA Mini Kit (Qiagen, Hilden, Germany) according to the manufacturer’s instructions. Briefly, 2 g of liver sample was homogenised on ice with the addition of 1 mL PBS using an Omni TH 220-PCRH mechanical tissue homogeniser (Omni International, Kennesaw, GA, USA), and centrifuged at 2–8 °C, 14,000× *g* for 5 min. The remaining supernatant was collected, and its portion was used for subsequent isolation of viral RNA. At this stage, a negative control of the nucleic acid isolation step that comprised all reagents and water instead of the sample was included for each batch of the analysed samples. Detection of HEV RNA was conducted using a single-step, real-time RT-qPCR with RNA UltraSense^TM^ One-Step Quantitative RT-PCR (Invitrogen, Carlsbad, CA, USA) as described previously [26]. The employed primers and probes targeted the ORF3 HEV genome [25]. Molecular detection was controlled by the incorporation of a target-specific internal amplification control (IAC) into the real-time PCR protocol, which constituted nucleic acid-containing primers’ sequences used in the real-time RT-qPCR [27]. The reactions were performed on the 7500 Real-Time PCR System (Applied Biosystems, Foster City, CA, USA). Apart from the IAC, the correct performance of the molecular detection step was also controlled by an appropriate negative (all reagents with distilled water instead of the template) and positive control, consisting of RNA derived from pig HEV gt 3 strain (accession number: MT770754). To determine the copy number of the HEV genome in liver tissues and faeces, a standard curve was prepared based on the RNA transcript and its 10-fold dilutions containing from 10^1^ to 10^5^ HEV G.C./μL.

### 2.6. Statistical Analysis

The seroprevalence values of HEV infections in wild boars, as well as the frequency of infections determined on the basis of molecular results, were estimated using the Clopper–Pearson method. Based on the estimated values of the confidence intervals for HEV seroprevalence, the regional (RDSF) differences in frequency of infection occurrence in animals were assessed using a chi-square (χ2) independence test for binomial distribution variables. The determined Spearman rank correlation coefficient values allowed the assessment of the credibility of the serological results. It was also used to show the relationship (correlation) between the serological (presence of antibodies) and molecular (presence of the virus in livers of seropositive animals) results. The adopted significance level was *p* = 0.05. All calculations were performed with a Statgraphics Centurion v. XV (Statpoint Technologies, Warrenton, VA, USA).

## 3. Results

### 3.1. Prevalence of Anti-HEV Antibodies in the Sera of Wild Boar

The presence of specific anti-HEV IgG antibodies was found in 232 (49.4%; 95%CI: 44.7–54%) out of 470 sera samples. Negative results were obtained for 224 (47.7%; 95%CI: 43.1–52.3%) sera, while 14 (3.0%; 95%CI: 47.7–56.9%) gave doubtful results. Seroprevalence above 60% was observed in RDSFs Zielona Góra (72%; 95%CI: 50.6–87.9%), Radom (66.7%, 95%CI: 29.9–92.5%) and Piła (65.2%, 95%CI: 42.7–83.6%) (Table 2). A significantly lower percentage of seropositive animals compared to the average country seroprevalence (49.4%; 95%CI: 44.7–54.0%) was found in RDSFs Kraków (10%, 95%CI: 0.2–44.5%) and Lublin (27%, 95%CI: 13.8–44.1%). Based on the χ2 test and confidence intervals estimated using the Clopper–Pearson method, the significant differences (χ2 = 27.7, *p* = 0.0344 < 0.05) in the seroprevalence of HEV infections were only found between RDSFs Zielona Góra, Kraków and Lublin. In the remaining regions, there were no significant differences observed in the frequency of infections in wild boars.

### 3.2. Detection of HEV RNA in Livers and Faeces

HEV RNA was detected in 57 (12.1%, 95%CI: 9.3–15.4%) out of 470 liver samples. The majority of actively infected animals were found in RDSF Katowice (11 wild boars, 95%CI: 13.5–41.2%) and 8 animals in each Olsztyn (95%CI: 8.8–34.9%) and Szczecin (95%CI: 6.7–27.6%) RDSF, respectively (Table 2). The viral load in liver tissues ranged from 1.4 to 1.7 × 10^11^ G.C./g (Table 3). In the case of animals originating from RDSFs Kraków, Warsaw and Wrocław, HEV RNA was not detected in their livers. The virus was also present in 27/433 (6.2%, 95%CI: 4.1–8.9%) faecal samples in the range of 38 to 9.3 × 10^7^ G.C./mL (Table 2 and Table 3). In the group of seropositive animals, there were individuals with virus-free livers, although HEV was present in animal faeces at high titres (up to 1.7 × 10^5^ G.C./mL). In contrast, in HEV-seronegative animals whose livers were PCR-negative, viral RNA was sporadically (2/224) detected in faeces.

When serological and molecular results for the pair of samples (serum and liver) were jointly analysed, a correlation (r = 0.560; *p* = 0.025) between the presence of specific anti-HEV antibodies in the sera and the virus in livers of seropositive individuals was observed. Nevertheless, the R^2^ determination coefficient for the observed relationship was 25%, which means that this correlation will not always be met.

## 4. Discussion

Wild boars are considered an important link in the epidemiology of HEV infections in humans and other animal species. In this study, the presence of anti-HEV antibodies in wild boars was found in 47.6% of animals, and this seroprevalence value was similar to that (44.4%) observed by Larska et al. [12] for central and western Poland. Additionally, in Europe, the seroprevalence of infections in wild boars ranged from 4.9% in Italy [28] to 57.4% in Spain [29]. A high percentage of infected wild boars was shown in Lithuania (25.9%) [30], Corsica (29.9%) [31], Slovenia (30%) [32], Belgium (34%) [33] and in the central part of Italy (56.2%) [34]. Regional differences in the occurrence of the frequency of HEV infections in wild boars in Poland were observed. Differing HEV infection rates (54.2%) were recorded in animals inhabiting the western rather than the eastern part of Poland (41.1%). The highest seroprevalence was found in RDSFs Radom (66.7%), Olsztyn (56%) and Białystok (55.6%), which have the largest population of wild boars in eastern Poland. However, the lowest level of anti-HEV antibodies, not exceeding 20% of animals in the population, was recorded in RDSFs Łódź and Kraków. Surprisingly, although RDSF Warsaw belonged to the regions with the lowest density of animals, it showed a high infection rate (46.7%). A high number of HEV-positive wild boars detected in Poland and some European countries can, to some extent, be explained by the varied number of animals tested in the population, the frequency of virus occurrence in a particular area, as well as by an increasing population of animals observed over the years [29,34,35]. For example, in Poland, the number of wild boars has increased 2.5 times from 118,300 in 2000 to 284,600 in 2014 [36]. It is evident that also in Germany and Spain, the number of seropositive animals rose over 10 years by 15.1% and 30.9%, respectively [9,29,37,38]. The differences in disease prevalence may also result from different sensitivity and specificity of the serological tests used. The currently employed ELISA-based assays utilise virus antigens derived from different virus genotypes. In this light, the need for standardisation of the serological tests used for the assessment of HEV seroprevalence in animals has been proposed [39]. Although the correlation coefficient between the serological (sera) and molecular results (liver testing) appeared to be significant, the virus will likely also be detected in the livers of seropositive animals. This relationship was only observed for 32.3% (44/136) of seropositive individuals with high (>140%) antibody titres. In this study, aside from sera and liver samples, wild boar faeces were also tested for HEV RNA. It was mainly found in the faeces of seropositive animals. Surprisingly, the faeces of some seronegative wild boars, whose livers gave negative results during molecular testing, also tested positive for the virus. It could be explained by an early stage of infection when the virus efficiently replicated in the intestinal epithelial cells before it reached the liver. An interesting observation was an absence of the virus in the livers of some seropositive wild boars despite its presence in the faeces. This may indicate virus clearance from the body after an acute stage of disease or its active replication in extrahepatic tissues long after it has disappeared from the liver. This assumption could be supported by a high virus titre (10^4^ G.C.) detected in the faeces. Nevertheless, the virus was found less frequently in the faeces than in the livers of infected animals. This study has two limitations: (i) an unknown specificity of ELISA used in this study for the detection of different virus genotypes, and (ii) a limited number of tested animals within each RDSF. Currently available ELISA kits are mainly intended for detecting HEV infections in humans and pigs. Unfortunately, there are no dedicated serological tests for any other animal species, whereby diagnostics of HEV infections in wild boars are carried out using tests developed for pigs. This is common laboratory practice, justified by a high antigenic relationship observed between the HEV gt 3 strains detected in these animal species [40,41]. In order to reflect the real seroprevalence of infections caused by strains of HEV gt 4, 5 and 6, future tests should employ, for antibody capture, a mixture of several antigenic components representing different virus genotypes. In this study, the number of tested sera was representative of the investigated animal population; therefore, the estimated seroprevalence of HEV infections in wild boars in Poland can be considered as credible. Contrary to this result, the seroprevalence values estimated for particular Polish RDSFs did not meet the assumption of the results characterised by 95% probability due to the low number of animals tested in each RDSF. Nevertheless, a high, significant value of the correlation coefficient (r = 0.944) between the size of the wild boar population and the number of tested sera samples obtained from each RDSF confirmed compliance with the principle of proportionality during sampling.

## 5. Conclusions

The results of this study provide comprehensive data on the occurrence of HEV infections in the population of wild boars in Poland. HEV infections in this animal species appeared to be common with the highest seroprevalence observed in this part of Europe. A correlation between serological and molecular results of testing of wild boars infected with HEV was shown. In order to get a better understanding about the zoonotic features of detected wild boar HEV strains and their epidemiological importance for public health, further studies are necessary for determining virus subtypes.

## Figures and Tables

**Table 1:** The population size of wild boars, their inhabiting area and the number of tested animals.

Regional Directorates of State Forest (RDSF)	Province	Population Size	Number of Animals
Katowice	Opole, Silesia	20–30,000	43
Olsztyn	Warmia-Masuria		41
Szczecin	Lubuskie, West Pomerania		53
Szczecinek	Pomerania, West Pomerania		44
Białystok	Podlaskie, Warmia-Masuria	10–20,000	21
Gdańsk	Pomerania, Warmia-Masuria		22
Krosno	Podkarpackie		17
Lublin	Lublin, Podkarpackie		37
Łódź	Łódź, Mazovia		17
Piła	Wielkopolska		23
Poznań	Wielkopolska		38
Radom	Mazovia, Świętokrzyskie		9
Toruń	Kujawy-Pomerania		30
Wrocław	Lower Silesia		25
Zielona Góra	Lubuskie		25
Kraków	Małopolska	<10,000	10
Warszawa	Mazovia		15
Total			470

**Table 2:** Results of serological and molecular testing of wild boars.

RDSF	No. of Sample	ELISA (%; 95%CI)				Liver (%; 95%CI)				No. of Sample	Faeces (%; 95%CI)			
		Positive		Negative		Positive		Negative			Positive		Negative	
Białystok	21 *	11	(52.4; 29.8–74.3)	9	(42.9; 21.8–66.0)	2	(9.5; 1.2–30.4)	19	(90.5; 69.6–98.8)	21	0	(0; 0–16.1)	21	(0; 83.8–100)
Gdańsk	22	11	(50.0; 28.2–71.8)	11	(50.0; 28.2–71.8)	4	(18.2; 5.2–40.3)	18	(81.1; 59.7–94.8)	22	0	(0; 0–15.4)	22	(0; 84.5–100)
Katowice	43	23	(53.5; 37.6–68.8)	20	(46.5; 31.2–62.3)	11	(25.6; 13.5–41.2)	32	(74.4; 58.8–86.5)	40	1	(2.5; 0–13.1)	39	(97.5; 86.8–99.9)
Kraków	10	1	(10.0; 0.2–44.5)	9	(90.0; 55.5–99.7)	0	(0; 0–30.8)	10	(100; 69.1–100)	10	0	(0; 0–30.8)	10	(0; 69.1–100)
Krosno	17	5	(29.4; 10.3–56)	12	(70.6; 44.0–89.7)	1	(5.9; 0.2–28.7)	16	(94.1; 71.3–99.8)	16	2	(12.5; 1.5–38.3)	14	(87.5; 61.6–98.4)
Lublin	37 *	10	(27.0; 13.8–44.1)	24	(64.9; 47.5–79.8)	1	(2.7; 0.1–14.2)	36	(97.3; 85.8–99.9)	35	0	(0; 0–10)	35	(0; 89.9–100)
Łódź	17	6	(35.3; 14.2–61.7)	11	(64.7; 38.3–85.8)	1	(5.9; 0.2–28.7)	16	(94.1; 71.3–99.8)	16	1	(6.2; 0.1–30.2)	15	(93.7; 69.7–99.8)
Olsztyn	41 *	23	(56.1; 39.7–71.5)	17	(41.5; 26.3–57.9)	8	(19.5; 8.8–34.9)	33	(80.5; 65.1–91.2)	38	4	(10.5; 2.9–24.8)	34	(89.4; 75.1–97)
Piła	23 *	15	(65.2; 42.7–83.6)	7	(30.4; 13.2–52.9)	3	(13; 2.8–33.6)	20	(87; 66.4–97.2)	22	2	(9.1; 1.1–29.1)	20	(90.9; 70.8–98.8)
Poznań	38	18	(47.4; 31–64.2)	20	(52.6; 35.8–69.0)	5	(13.2; 4.4–28.1)	33	(86.8; 71.9–95.6)	30	1	(3.3; 0–17.2)	29	(96.6; 82.7–99.9)
Radom	9	6	(66.7; 29.9–92.5)	3	(33.3; 7.5–70.1)	4	(44.4; 13.7–78.8)	5	(55.6; 21.2–86.3)	9	3	(33.3; 7.4–70)	6	(66.6; 29.9–92.5)
Szczecin	53 *	27	(50.9; 36.8–64.9)	23	(43.4; 29.8–57.7)	8	(15.1; 6.7–27.6)	45	(84.9; 72.4–93.3)	45	2	(4.4; 0.5–15.1)	43	(95.5; 84.5–99.4)
Szczecinek	44 *	22	(50.0; 34.6–65.4)	21	(47.7; 32.5–63.3)	3	(6.8; 1.4–18.7)	41	(93.2; 81.3–98.6)	40	1	(2.5; 0–13.1)	39	(97.5; 86.8–99.9)
Toruń	30 *	16	(53.3; 34.3–71.7)	12	(40.0; 22.7–59.4)	5	(16.7; 5.6–34.7)	25	(83.3; 65.3–94.4)	27	5	(18.5; 6.3–38)	22	(81.4; 61.9–93.7)
Warszawa	15 *	7	(46.7; 21.3–73.4)	7	(46.7; 21.3–73.4)	0	(0; 0–21.8)	15	(100; 78.2–100)	15	0	(0; 0–21.8)	15	(0; 78.1–100)
Wrocław	25 *	13	(52.0; 31.3–72.2)	11	(44.0; 24.4–65.1)	0	(0; 0–13.7)	25	(100; 86.3–100)	25	3	(12.5; 2.6–32.3)	21	(87.5; 67.6–97.3)
Zielona Góra	25	18	(72.0; 50.6–87.9)	7	(28.0; 12.1–49.4)	1	(4; 0.1–20.3)	24	(96; 79.7–99.9)	23	2	(8.7; 1–28)	21	(91.3; 71.9–98.9)
Total (%; 95%CI)	470 (100)	232 (49.4; 44.7–54)		224 (47.7; 43.1–52.3)		57 (12.1; 9.3–15.4)		413 (87.9; 84.6–90.7)		433 (100)	27 (6.2; 4.1–8.9)		406 (91.3; 91–93.7)	

* The numbers also include ELISA doubtful samples. They were not taken into account in the estimation of confidence intervals for positive and negative results.

**Table 3:** Results of molecular and quantitative analysis of liver and faeces of wild boars.

Number of Animals *	ELISA Results	Real-Time (RT)-qPCR Results	
	Serum	Liver (G.C./g)	Faeces (G.C./mL)
16	+	1.6 × 10^3^–9.4 × 10^9^	1.2 × 10^3^–9.3 × 10^7^
31 (1)	+	8.9–1.7 × 10^11^	-
5	+	-	38–1.7 × 10^5^
180 (8)	+	-	-
4	−	3.7–7.1 × 10^8^	8 × 10^2^–3.1 × 10^6^
11 (3)	−	1.4–1.1 × 10^9^	-
2	−	-	42–3.9 × 10^4^
207 (27)	−	-	-
14	+/−	-	-

(+)—positive result; (−)—negative result; (+/−)—doubtful results; * the number of animals for which only liver samples were tested are indicated in the brackets.
